# Musculoskeletal Manifestations of Sickle Cell Anaemia: A Pictorial Review

**DOI:** 10.1155/2011/794283

**Published:** 2011-02-06

**Authors:** A. Ganguly, W. Boswell, H. Aniq

**Affiliations:** Department of Radiology, Royal Liverpool University Hospital, Prescot Street, Liverpool L7 8XP, UK

## Abstract

Sickle cell anaemia is an autosomal recessive genetic condition producing abnormal haemoglobin HbS molecules that result in stiff and sticky red blood cells leading to unpredictable episodes of microvascular occlusions. The clinical and radiological manifestations of sickle cell anaemia result from small vessel occlusion, leading to tissue ischemia/infarction and progressive end-organ damage. In this paper we discuss and illustrate the various musculoskeletal manifestations of sickle cell disease focusing primarily on marrow hyperplasia, osteomyelitis and septic arthritis, medullary and epiphyseal bone infarcts, growth defects, and soft tissue changes.

## 1. Introduction

Sickle cell anaemia is an autosomal recessive genetic condition due to a mutation in the beta-globin gene resulting in replacement of glutamic acid in position 6 of the beta-globin chain by valine resulting in an abnormal haemoglobin HbS molecule. The term sickle cell disease applies to those patients who have at least one abnormal HbS chain and another abnormal beta chain. If the second abnormal chain is also an HbS chain then the patient is considered to be homozygous Hb SS-defined as sickle cell anaemia. Alternatively, other abnormal haemoglobin chains like Hb C or thalassemia result in Hb SC and Hb S-thal, respectively. The combination of an abnormal HbS chain and a normal beta-globin chain is called sickle cell trait. 

 The abnormal HbS protein chain polymerizes reversibly in deoxygenated environment into a gelatinous network of fibrous polymers that stiffen the RBC membrane, increases the viscosity, and causes dehydration resulting in a sickle shape. These abnormal cells lose there pliability and are abnormally sticky provoking unpredictable episodes of microvascular occlusions and premature haemolysis. The clinical and radiological manifestations of sickle cell anaemia are manifold; however, pathophysiologically all of them result from rigid adherent cells cogging small vessels, leading to tissue ischemia/infarction and gradual end-organ damage. 

In this paper we discuss and illustrate the various musculoskeletal manifestations of sickle cell disease. For the benefit of the reader we have subdivided the paper into the following sections.

## 2. Marrow Replacement and Hyperplasia

**Figure 1:**
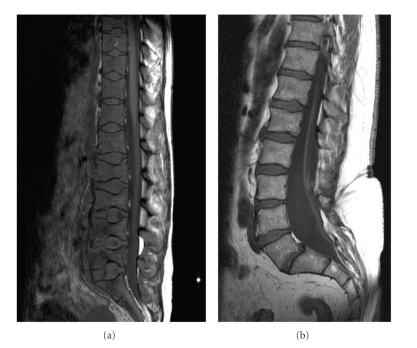
(a) shows a T1W sagittal MRI spine of a patient with sickle cell anaemia with diffuse low signal from the vertebral bodies consistent with hyperplastic haematopoietic red marrow replacing the normal bright fatty marrow. Note smooth concavity of the vertebral endplates at multiple levels from bone softening (fish mouth vertebra). Normal appearance of marrow is depicted on (b) for comparison.

**Figure 2:**
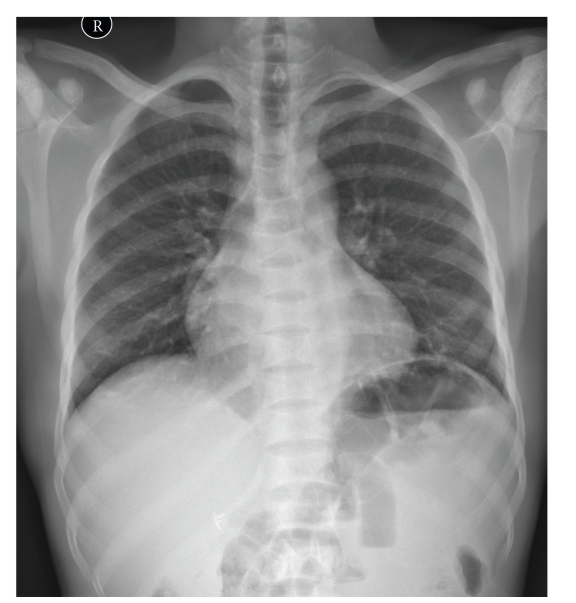
Chest radiograph showing coarsening of trabecular pattern with loss of corticomedullary differentiation of the ribs subject to haematopoietic marrow replacement in a patient with sickle cell disease. Note avascular necrosis involving the humeral heads bilaterally and colonic gas replacing splenic shadow in the left upper quadrant (secondary sign of autosplenectomy/small spleen subject to previous infarcts).

In a normal healthy adult haematopoietic red marrow is found in the axial skeleton (spine, sternum, pelvis, ribs, and proximal long bones) with yellow marrow conversion in the rest of the appendicular skeleton. As the sickle cell patient is chronically anaemic there is persistence of red marrow in both the axial and appendicular skeleton into adulthood together with bone marrow hyperplasia. On T1W MRI, normal fatty marrow shows high signal intensity, while haematopoietic red marrow is low in signal (Figures 1(a) and 1(b)). Marrow hyperplasia results in widening of the medulla and subsequent cortical thinning, resulting in coarsening of the normal trabecular pattern with loss of corticomedullary differentiation in both long and flat bones (Figure 2). This process may also cause the bone to appear osteopaenic and make the bone prone to softening and fracture [1]. The best example of bone softening is seen in the vertebral bodies, where the end plates assume a smooth concavity described as fish mouth vertebra (Figure 1(a)). 

Although less common in sickle cell disease, widening of the diploic space in the skull results in “hair on end” appearances, which is classically described in other forms of haemoglobinopathy-like thalassemia [2]. 

A further form of marrow hyperplasia is extramedullary haematopoiesis (although this is relatively uncommon in sickle cell anaemia, being more usually associated with sickle cell variants like HbS-thal, hereditary spherocytosis and thalassemia) [3]. Potential sites include the liver and spleen and paraspinal soft tissues. Rarer locations include the middle ear [4]. Radionuclide imaging with sulphur colloid imaging can confirm the haematopoietic nature of these masses [5]. Foci of extramedullary haematopoiesis are seen as well defined focal mass lesions and show intermediate signal weighting on T1 and T2 weighted MR images and are of soft tissue attenuation at CT [1].

Patients with sickle cell anaemia who have received multiple transfusions may develop secondary haemosiderosis, with excess iron collecting in the reticuloendothelial system. Classically it shows diffuse low signal on T2W gradient echo images, involving the liver and spleen, subject to the increased sensitivity of GRE images to magnetic susceptibility artefact of Iron.

## 3. Osteomyelitis

Osteomyelitis occurs in 18% while septic arthritis occurs in 7% of patients with sickle cell disease according to a study [6]. Patients with sickle cell anaemia have an increased incidence of septic arthritis and osteomyelitis as compared to the general population due to the abnormal red blood cells reducing flow in the small vessels, resulting in relative ischemic zones [7]. The body's own immunological response is less effective in areas of impaired vascularity. Hyposplenism due to autosplenectomy also results in a degree of immunocompromise. Osteomyelitis is most common in the diaphyses of long bones [8]. There is an increased incidence of salmonella osteomyelitis in sickle cell patients [9], where it is believed to be the most common pathogen: staph. aureus being the second most common organism [9]. 

The classical clinical findings of pain, fever, and raised inflammatory markers can also be seen in infarction, which can cause diagnostic difficulty [3]. 

**Figure 3:**
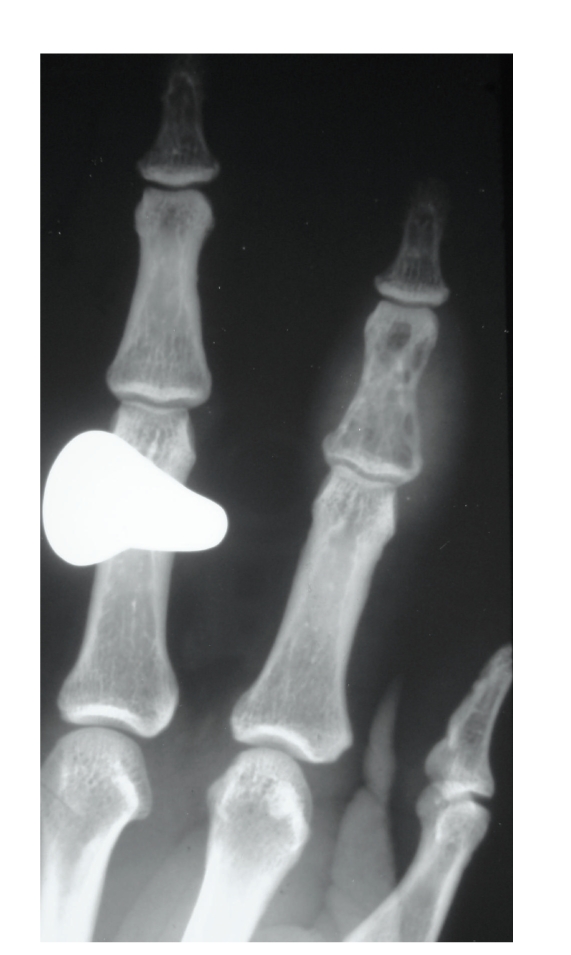
Plain radiograph of the hand of a patient with sickle cell anaemia showing patchy lucency and associated soft tissue swelling consistent with osteomyelitis of the middle phalanx of the index finger.

**Figure 4:**
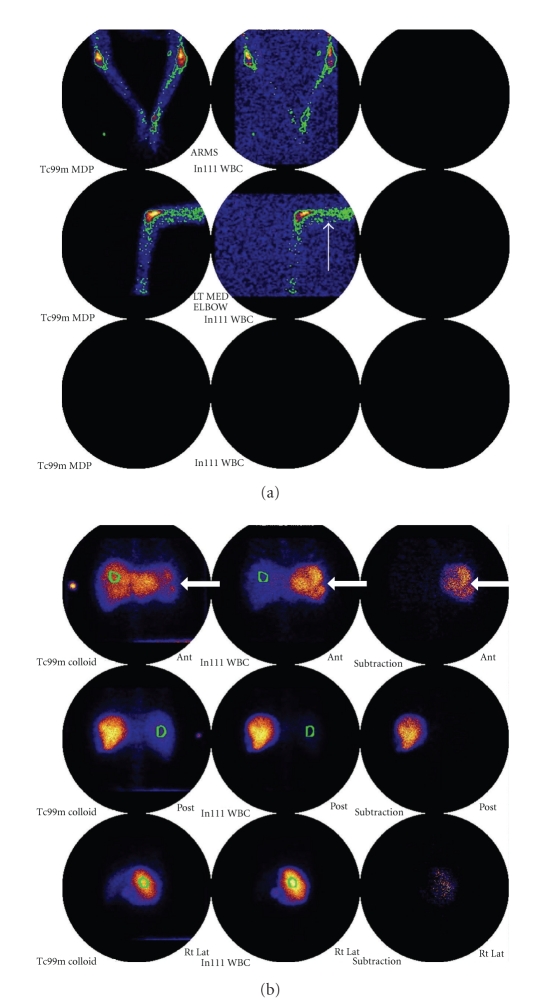
(a) In111 WBC and Tc99m colloid scan images in a patient with sickle cell anaemia showing abnormal increased activity in the distal left humerus on the WBC scan (white arrows) consistent with osteomyelitis. (b) Note also that multiple photopenic areas in the splenic tissue, seen on both WBC and colloid images (thick white arrows) suggest multiple splenic infarcts.

Plain film findings of osteomyelitis include osteopaenia, periosteal reaction with or without associated cortical destruction, sinus tract formation, and soft tissue extension (Figure 3). Features such as osteopaenia and periosteal reaction are not specific to osteomyelitis and can also be seen in acute bone infarction. As in osteomyelitis in nonsickle cell patients, the plain film findings lag behind the clinical picture and the plain film may be normal for up to 10 days [1]. Isotope bone scan and labelled white cell scans may be helpful, with triple phase bone scan showing increased activity in all 3 phases [1]. Labelled white cell scans can show increased uptake in infection with reduced uptake in areas of infarction, but the diffuse marrow abnormality present can hinder interpretation (Figure 4) [10, 11].

MR scanning is the preferred method of assessment [3]. On T2 weighted images, areas of osteomyelitis will be of increased signal intensity [12]. On T1 weighting, osteomyelitis is of low signal intensity (although areas of red marrow will also be of low signal intensity). Focal fluid collections and associated soft tissue abnormalities can also be demonstrated. Osteomyelitis will also show areas of enhancement postgadolinium. This will tend to be more diffuse than in infarction. Rim enhancement may also be seen but is not specific as it is also seen in bone infarction [13]. Soft tissue enhancement is again not specific for infection and can be seen in both. 

**Figure 5:**
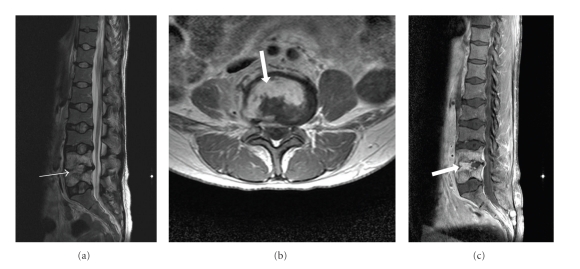
(a), (b), and (c) T2W sagittal, T1W axial and sagittal postgadolinium images in a patient with sickle cell anaemia, showing high signal in the anterior aspect of the L4/5 disc on T2W sequence (thin arrow) and enhancement on the post contrast images (thick arrows) consistent with discitis.

In the spine particularly, high signal is seen on T2 weighted images in the affected disc or vertebral body in the case of infective discitis and vertebral osteomyelitis with enhancement of the abnormal areas postgadolinium injection (Figures 5(a), 5(b) and 5(c)).

As well as osteomyelitis, patients with sickle cell disease have an increased incidence of septic arthritis [1]. Joint effusion will be seen (although this can also be seen with bone infarction). Ultrasound may be used to confirm or refute the presence of effusion and also to guide joint aspiration. MR may also demonstrate joint fluid. Synovium enhances vividly postgadolinium and there may be bone marrow oedema surrounding the joint.

## 4. Bone Infarcts: Epiphyseal and Medullary

Abnormal red cell shape blocking capillaries result in bone infarction in both the diaphyses, causing medullary infarcts and in the epiphyses, causing the appearances of avascular necrosis. This can present as the classical painful bone crisis. 

**Figure 6:**
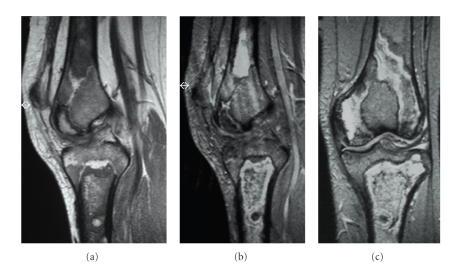
(a), (b) and (c) T1W sagittal, T2W sagittal, and coronal MRI knee in a patient with sickle cell anaemia showing, low signal change on T1W images with corresponding areas of solid and serpiginous high signal change on T2W sequence consistent with medullary infarcts involving the femur and tibia.

Medullary bone infarcts are far more common than osteomyelitis in patients with sickle cell disease [14], but clinical differentiation can be difficult [7]. Initial radiographs are usually normal with an acute infarction [12]. Later films show patchy lucency, possibly with periosteal reaction [3]. As the condition becomes more chronic sclerosis develops. Bone scintigraphy may show initial photopenia, but as the bone revascularises uptake may return to normal or even be increased. This makes interpretation of the radioisotope imaging difficult as the relevance of the imaging findings is related to the length of time since the infarction [1]. On T2 weighted MR scanning, infarction is seen as an area of high signal intensity (as in osteomyelitis) [13]. Infarcts may also show peripheral enhancement postgadolinium and soft tissue change, further complicating diagnosis [15] (Figures 6(a), 6(b), and 6(c)).

**Figure 7:**
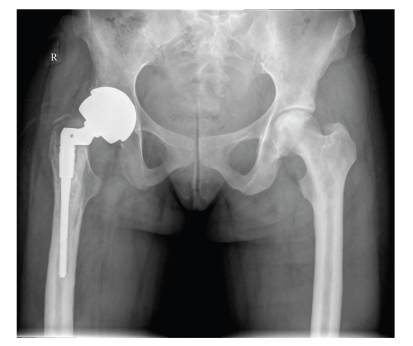
Plain radiograph of the pelvis showing sclerosis of the left femoral head consistent with avascular necrosis. Note total hip replacement due to premature secondary osteoarthritis secondary to avascular necrosis on the right.

**Figure 8:**
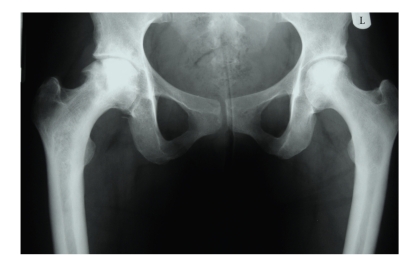
Plain radiograph of the pelvis in another patient with sickle cell anaemia more advanced avascular necrosis with sclerosis and subchondral collapse of the right femoral head. Less severe changes are seen on the left.

**Figure 9:**
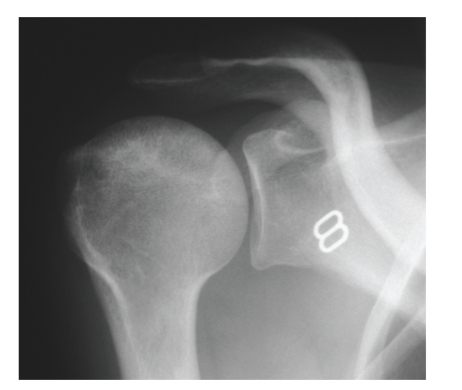
Plain radiograph of the shoulder showing early patchy sclerosis of the right humeral head consistent with early avascular necrosis (snow storm appearance).

**Figure 10:**
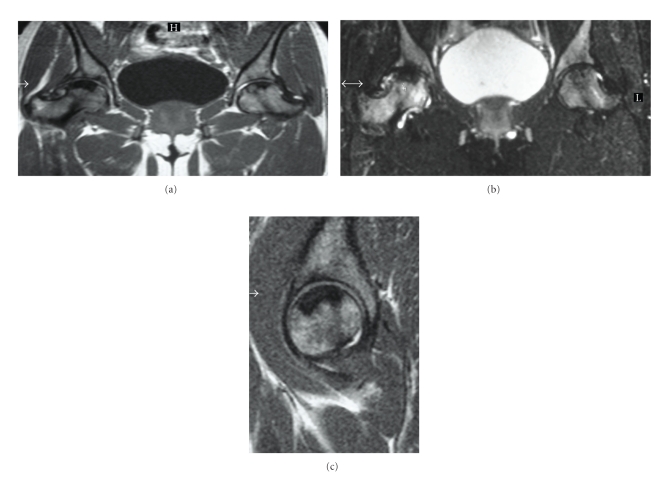
(a), (b), and (c) T1W coronal, T2FS (fat saturated) coronal and T1W sagittal MRI of the pelvis and right hip of a patient with sickle cell anaemia. Advanced avascular necrosis with subchondral collapse is seen superiorly in the femoral heads as evidenced by low signal areas on both T1W and T2W images. Additional areas of high signal, particularly in the right femoral neck on the T2FS images depict further areas of ischemia.

A further pattern of bone infarction is involving the epiphysis. This is more usually referred to as avascular necrosis (Figures 2, 7, 8, and 9). Again, initial radiographs are normal. Later typical appearances of sclerosis, subchondral collapse, and flattening can be appreciated. It is stated that about 40% of patients with sickle cell disease would develop AVN by their mid 50s [16]. T2 weighted MR scanning shows high signal intensity (fat suppressed sequences are particularly sensitive). A serpiginous low signal intensity line is classically seen. As the necrosis progresses, sclerosis develops, and there is collapse of the affected epiphysis (Figures 10(a), 10(b), and 10(c)).

**Figure 11:**
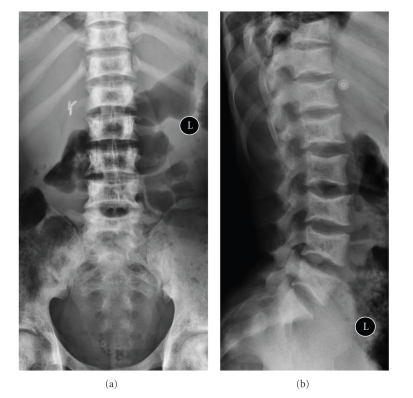
(a) and (b) AP and lateral plain radiographs of the lumbar spine showing sharp end plate depressions due to central end-plate infarction resulting in classic H-shaped vertebrae. On the other hand bone softening results in smooth concavity described as fish mouth vertebra. Also note cholecystectomy clips from previous surgery for pigmented stones and patchy sclerosis of the pelvic bones from medullary infarction.

In the spine, AVN of the end plate produces a sharp central step in the vertebral body end plate, causing the H-shaped vertebra (Figures 11(a) and 11(b)). This can be easily differentiated from the smooth concavity seen with bone softening.

In children, infarction within the small bones of the hand and feet result in painful dactylitis termed “hand-foot” syndrome; as red marrow does not persist in the hands and feet into later childhood, the syndrome is not seen in children beyond the age of 5 [17]. It presents with painful extremities and radiologically shows soft tissue swelling, periosteal new bone, and patchy areas of sclerosis and lucency [1].

## 5. Soft Tissue Abnormalities

Occlusion of vessels leads to inflammation and myonecrosis resulting in areas of fluid collection, haematoma, infarction, or abscess formation in the muscles. Abnormal areas show high signal on T2 weighted and fluid sensitive sequences on MRI. Areas of infection can occur independently or in conjunction with osteomyelitis where it again shows high signal on T2 weighted images and postgadolinium ring enhancement [1]. 

Leg ulcers are also common, particularly over bony prominences subject to tissue ischemia.

## 6. Growth Effects

Patients with sickle cell anaemia have reduced height [18]. This is believed to be due to bone marrow hyperplasia [7]. Bones are generally shorter due to epiphyseal shortening subject to ischemia/infarction and vascular compromise to the growth plate. Premature closure of growth plates also occurs.
